# *Pf*SRPK1 Regulates Asexual Blood Stage Schizogony and Is Essential for Male Gamete Formation

**DOI:** 10.1128/spectrum.02141-22

**Published:** 2022-09-12

**Authors:** Sudhir Kumar, Vinay K. Baranwal, Amanda S. Leeb, Meseret T. Haile, Kenza M. Z. Oualim, Nina Hertoghs, Ashley M. Vaughan, Stefan H. I. Kappe

**Affiliations:** a Center for Global Infectious Disease Research, Seattle Children’s Research Institute, Seattle, Washington, USA; b Molecular Botany Lab, Swami Devanand Post Graduate College, Math-Lar, Deoria, Uttar Pradesh, India; c Department of Global Health, University of Washington, Seattle, Washington, USA; d Department of Pediatrics, University of Washington, Seattle, Washington, USA; University of Illinois at Urbana-Champaign

**Keywords:** SRPK1, gametocyte, exflagellation, mosquito, transmission, RNA-seq

## Abstract

Serine/arginine-rich protein kinases (SRPKs) are cell cycle-regulated serine/threonine protein kinases and are important regulators of splicing factors. In this study, we functionally characterize SRPK1 of the human malaria parasite Plasmodium falciparum. P. falciparum *SRPK1* (*PfSRPK1*) was expressed in asexual blood-stage and sexual-stage gametocytes. *Pfsrpk1*^−^ parasites formed asexual schizonts that generated far fewer merozoites than wild-type parasites, causing reduced replication rates. *Pfsrpk1*^−^ parasites also showed a severe defect in the differentiation of male gametes, causing a complete block in parasite transmission to mosquitoes. RNA sequencing (RNA-seq) analysis of wild-type *Pf*NF54 and *Pfsrpk1*^−^ stage V gametocytes suggested a role for *Pf*SRPK1 in regulating transcript splicing and transcript abundance of genes coding for (i) microtubule/cilium morphogenesis-related proteins, (ii) proteins involved in cyclic nucleotide metabolic processes, (iii) proteins involved in signaling such as *Pf*MAP2, (iv) lipid metabolism enzymes, (v) proteins of osmophilic bodies, and (vi) crystalloid components. Our study reveals an essential role for *Pf*SRPK1 in parasite cell morphogenesis and suggests this kinase as a target to prevent malaria transmission from humans to mosquitoes.

**IMPORTANCE** *Plasmodium* sexual stages represent a critical bottleneck in the parasite life cycle. Gametocytes taken up in an infectious blood meal by female anopheline mosquito get activated to form gametes and fuse to form short-lived zygotes, which transform into ookinetes to infect mosquitoes. In the present study, we demonstrate that *Pf*SRPK1 is important for merozoite formation and critical for male gametogenesis and is involved in transcript homeostasis for numerous parasite genes. Targeting *Pf*SRPK1 and its downstream pathways may reduce parasite replication and help achieve effective malaria transmission-blocking strategies.

## INTRODUCTION

The Plasmodium falciparum malaria life cycle alternates between human hosts and female *Anopheles* mosquitoes. Within the human host, the parasites proliferate in red blood cells through schizogony. Asexual schizonts undergo cell growth and multiple rounds of DNA replication to generate invasive daughter merozoite forms, which are released and infect new red blood cells. A small proportion of asexually replicating parasites commit to the formation of transmissible sexual forms called gametocytes. Gametocytes undergo an approximately 2-week-long maturation process within the infected cell, developing through five morphologically distinct stages (stages I to V) that involve cellular remodeling and are accompanied by membrane changes of the host red blood cell, transient deposition of surface antigens, and a reversible increase in cellular rigidity (1). When mature stage V gametocytes are taken up by the female mosquito in an infected blood meal, they are activated and undergo gametogenesis to form female (macro-) and male (micro-) gametes. While gametogenesis involves few morphological changes in the macrogamete after egress from erythrocytes, male gametogenesis involves three rounds of DNA replication and radical cellular changes leading to the assembly of eight axonemes in order to produce eight flagellated microgametes (2–4). These motile microgametes are released from red blood cells through a process known as exflagellation (4). When a microgamete encounters a macrogamete inside the blood meal, it attaches, and both cells undergo cell fusion to form a zygote. The zygote transforms into a motile ookinete that penetrates the midgut epithelium and develops into an oocyst, which ultimately produces sporozoites.

Gametogenesis is triggered by host environmental factors, including a temperature decrease after mosquito uptake, a pH change (5), and the presence of xanthurenic acid (XA), a metabolite of tryptophan (6). These factors then initiate signaling cascades that mediate the gametocyte-to-gamete transition. The cyclic GMP (cGMP)-dependent protein kinase, PKG, mediates the release of Ca^2+^ from intracellular stores (7), which activate Ca^2+^-dependent protein kinase 1 (CDPK1), CDPK2, and CDPK4, which are involved in male gametogenesis (8–10). The gametogenesis essential protein 1 (GEP1) has been identified for its role in XA-stimulated gametogenesis in Plasmodium yoelii (11). Other studies have identified cyclin-dependent kinase (CDK)-related kinase 5 (CRK5) as a critical regulator of endomitosis during male gametogenesis (12). Further kinases and phosphatases with roles in gametogenesis include a mitogen-activated protein kinase, MAP2 (13), metallo-dependent protein phosphatase 1 (PPM1) (14), and Ca^2+^-dependent calcineurin A (CnA) (15).

In most eukaryotes, protein kinase signaling cascades modulate gene expression by regulating transcriptional or posttranscriptional events (16, 17). The complex life cycle of the malaria parasite requires regulation of gene expression in the various developmental stages (18, 19). In addition to transcription factors, regulatory mechanisms such as translational repression of mRNAs (20) and control of gene expression by antisense RNA have also been described for *Plasmodium* (21, 22). Splicing of precursor mRNAs (pre-mRNAs) is one of the major regulatory mechanisms of eukaryotic gene expression. Alternative mRNA splicing (AS), which generates transcripts that encode structurally and functionally distinct protein isoforms, is a central mechanism for physiological regulation of proteome diversity (23) and has been observed for *Plasmodium* spp. (24–27). In eukaryotes, the serine/arginine-rich (SR) proteins are critical components of the splicing machinery (spliceosome), which regulates both constitutive and alternative splicing of pre-mRNA. The phosphorylation of SR proteins by CDC-like kinases (CLKs) (28) and closely related serine/arginine protein kinase (SRPKs) (29, 30) is a central regulatory mechanism for RNA splicing (31). SRPK1 phosphorylation of splicing factors is restricted by a specific docking interaction, whereas CLK activity is less constrained, with broader substrate specificity (32). While SRPKs are present in both nuclear and cytoplasmic compartments in the cell (33), their interaction with SR proteins is required for their translocation to the nucleus (34). Mammalian SRPK1 also regulates phosphorylation of non-SR proteins, including protamines, which trigger DNA decondensation and histone deposition postfertilization (35). *Plasmodium* genomes encode two members of the CLK protein kinase family, namely, CLK1 (PF3D7_1445400) and CLK3 (PF3D7_1114700), and two members of the SRPK family, SRPK1 (PF3D7_0302100) and SRPK2 (PF3D7_1443000) (PlasmoDB). Pharmacological inhibitors of *Pf*CLK3 have shown potent killing of P. falciparum liver stages and blood stages and blockade of gametocyte development and, thus, parasite transmission (36).

*PfSRPK1* was reported to be refractory to gene disruption (37), and the pharmacological inhibitors of *Plasmodium* CLKs/SRPKs indicate their important roles in asexual replication (36, 38). We here revisited the role of SRPK1 in P. falciparum asexual blood-stage replication and sexual-stage development. In contrast to previous findings, we were able to create *Pfsrpk1^−^* parasites using CRISPR/Cas9-based gene deletion. We show that *PfSRPK1* has a function in daughter merozoite formation during asexual blood-stage replication. Strikingly, we also demonstrate that although *PfSRPK1* is dispensable for sexual-stage commitment, it is essential for male gametogenesis and, thus, transmission to the mosquito vector. Comparative bulk transcriptomic analysis of wild-type *Pf*NF54 and the *Pfsrpk1^−^* stage V gametocytes using transcriptome sequencing (RNA-seq) showed perturbation of transcript splicing, significant downregulation of transcript abundance for cilium/microtubule-based movement-related proteins, and signaling proteins such as kinases, phosphatases, and proteins involved in cyclic nucleotide metabolism, as well as enzymes of fatty acid metabolism in the *Pfsrpk1^−^* parasites.

## RESULTS

### *Pf*SRPK1 is expressed in asexual and sexual parasite stages.

**FIG 1:**
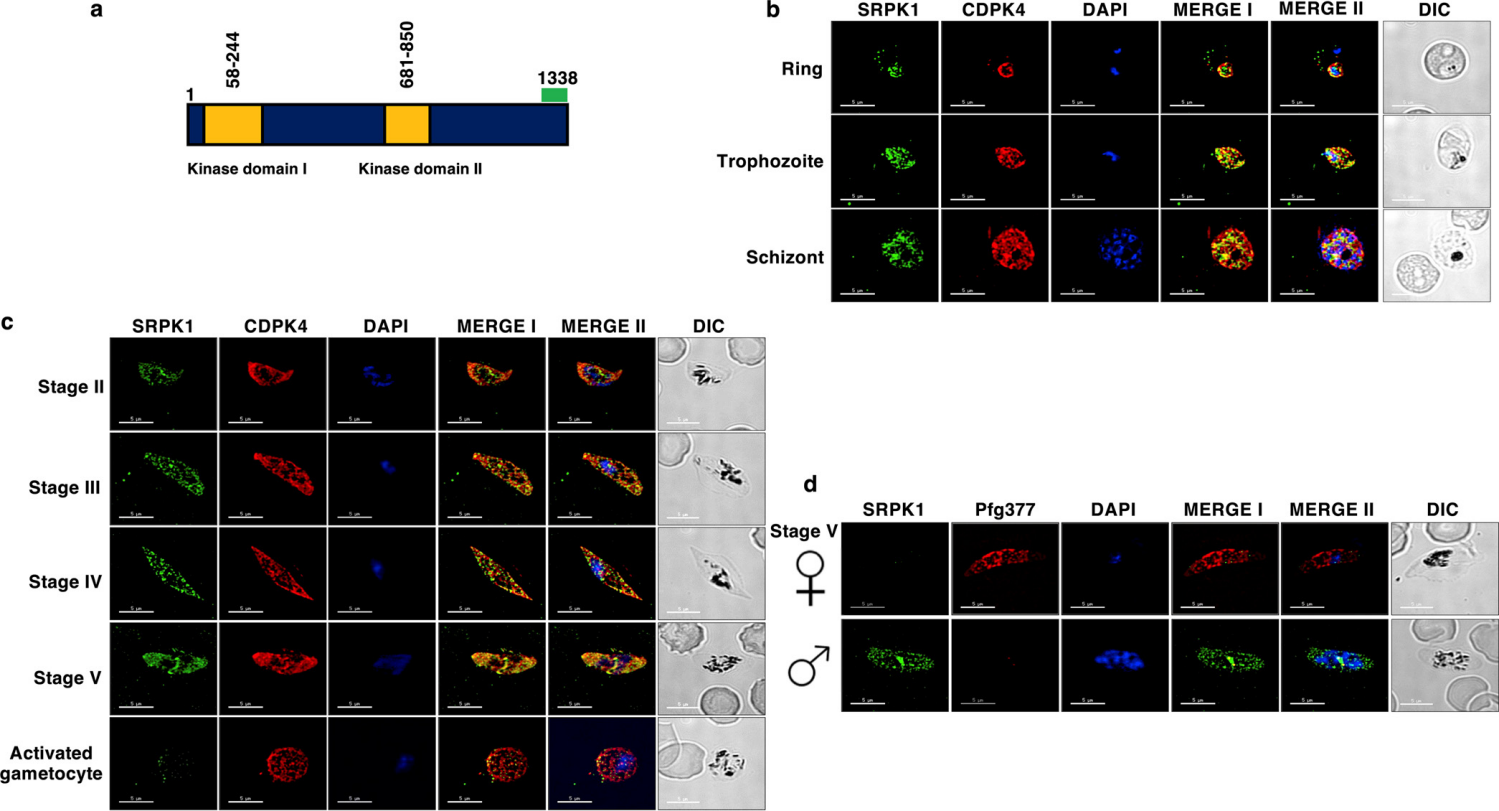
Expression and localization of *Pf*SRPK1 in asexual and sexual parasite stages. (a) Schematic for *Pf*SRPK1 protein shows that its single kinase domain has a spacer region dividing it into kinase domains I and II. The peptide region used for antisera generation is indicated in green. (b) IFAs were performed on asexual stages (ring, trophozoite, schizont) using thin culture smears and anti-*Pf*SRPK1 antisera (green) in combination with anti-*Pf*CDPK4 antibodies (red). The parasite DNA was visualized with DAPI (blue). Scale bar, 5 μm. Images are shown from representative experiments. Merge I, merged image for red and green channels; merge II, merged image for red, green, and DAPI (blue) channels. (c) IFAs were performed on sexual stages (stage II to V gametocytes) and 10 min postactivation using smears and anti-*Pf*SRPK1 antisera (green) in combination with anti-*Pf*CDPK4 (red). The parasite DNA was visualized with DAPI (blue). (d) IFAs were performed on stage V gametocytes using anti-*Pf*SRPK1 antisera (green) in combination with anti-*Pf*g377 antibodies (red; for female gametocytes). *Pf*SRPK1 staining was negative for female gametocytes. The parasite DNA was visualized with DAPI (blue). Scale bar, 5 μm. Representative images are shown. Merge I, merged image for red and green panels; merge II, merged image for red, green, and DAPI (blue) channels. DIC, differential interference contrast; DAPI, 4′,6-diamidino-2-phenylindole.

To analyze the expression of *Pf*SRPK1 in asexual and sexual parasite stage development, we generated antisera against a synthetic keyhole limpet hemocyanin (KLH)-conjugated peptide (LIENRDDQNVNKINCKVINKKNSC) based on the C-terminal amino acid sequence of the protein (Fig. 1a). Indirect immunofluorescence assays (IFAs) revealed that *Pf*SRPK1 is expressed in ring stages, trophozoite stages, and schizont stages (Fig. 1b). *Pf*SRPK1 expression was also detected in gametocytes from stage II through stage V (Fig. 1c). It appeared to be absent in activated gametocytes (Fig. 1c). *Pf*SRPK1 appeared to localize within the parasite cytoplasm of all stages for which expression was detected. Interestingly, dual labeling with anti-*Pf*CDPK4 antibody, which is a marker for both male and female gametocytes, and a female (anti-*Pf*g377) gametocyte-specific antibody revealed that *Pf*SRPK1 is expressed only in male gametocytes and is absent in female gametocytes (Fig. 1c and d), suggesting a male gender-specific function for *Pf*SRPK1.

### *Pf*SRPK1 is important for asexual blood-stage replication.

**FIG 2:**
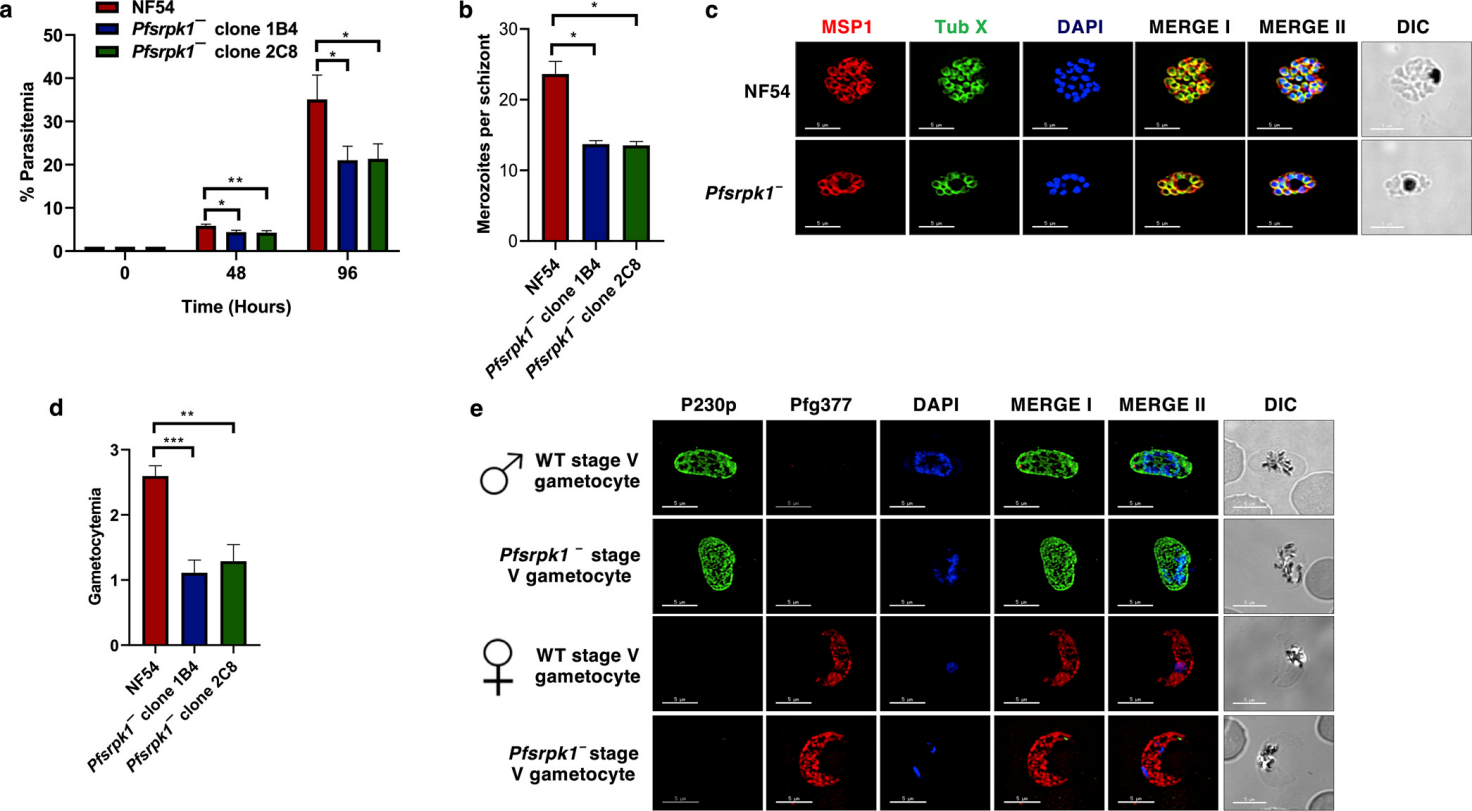
Asexual blood-stage and sexual-stage phenotypes of *Pfsrpk1^−^* parasites. (a) Percentage of parasitemia showing the increase in parasitemia in two subsequent generations of WT *Pf*NF54 and *Pfsrpk1^−^* (clones 1B4 and 2C8). *Pfsrpk1^−^* parasites showed reduced growth compared to WT *Pf*NF54. The means ± standard deviations (SDs) (error bars) of three biological replicates are shown. (b) Number of nuclei per schizont in WT *Pf*NF54 and *Pfsrpk1^−^* parasites (clones 1B4 and 2C8). (Duplicate experiments; *n* = 50 cells for each condition; bars are SDs). (c) IFAs were performed on mature schizont stages for WT *Pf*NF54 and *Pfsrpk1^−^* using thin culture smears with anti-*Pf*MSP1 antisera, which labels the merozoite surface (red) in combination with anti-tubulin X antibodies, which would mark the subpellicular microtubules (green). Representative images are shown. The parasite DNA was visualized with DAPI (blue). Scale bar, 5 μm. Merge I, merged image for red and green panels; merge II, merged image for red, green, and DAPI (blue) channel. DIC, differential interference contrast; DAPI, 4′,6-diamidino-2-phenylindole. (d) Gametocytemia for WT *Pf*NF54 and *Pfsrpk1^−^* parasites (clones 1B4 and 2C8) was measured on day 15 using Giemsa-stained thin culture smears. *Pfsrpk1^−^* gametocytes showed reduced gametocytemia compared to WT *Pf*NF54. The means ± SDs (error bars) of three biological replicates are shown. (e) IFAs were performed on WT *Pf*NF54 and *Pfsrpk1^−^* mature stage V gametocytes culture thin smears using anti-*Pf*P230p antisera, which labels the stage V male gametocytes (green) in combination with anti-*Pf*g377 antisera, which labels female gametocytes (red). Representative images are shown. The parasite DNA was visualized with DAPI (blue). Scale bar, 5 μm. Merge I, merged image for red and green panels; merge II, merged image for red, green, and DAPI (blue) channel. Symbols for male and female gametocytes are shown on the left sides of the image panels.

Previous studies have claimed that *Pf*SRPK1 is an essential gene for asexual blood-stage development because it is refractory to gene disruption (37). However, we were able to delete *PfSRPK1* using a CRISPR/Cas9 strategy. DNA regions upstream and downstream of the *PfSRPK1* locus were PCR amplified and ligated through an overlapping linker and cloned into the pFCL3 vector (see Fig. S1a in the supplemental material), which has previously been used for gene editing in P. falciparum (39). Two 20-nucleotide guide sequences were also cloned into the vector to create two separate plasmids, which were mixed and transfected into wild-type (WT) *Pf*N54 parasites. Recombinant parasites were drug selected, PCR genotyped, and then cloned by limiting dilution. The *PfSRPK1* locus deletion (*Pfsrpk1^−^*) in clonal parasites was confirmed by a set of genotyping PCRs (Fig. S1b and c). IFAs performed on *Pfsrpk1^−^* parasites using anti-*Pf*CDPK4 and anti-*Pf*SRPK1 antibodies showed no *Pf*SRPK1 staining, further confirming the absence of *Pf*SRPK1 protein and also the specificity of the antisera (Fig. S1d). To identify a potential effect of the *PfSRPK1* deletion on asexual parasite replication, we performed *in vitro* culture growth assays comparing WT *Pf*NF54 and *Pfsrpk1^−^* parasites (clones 1B4 and 2C8) over two replication cycles, enumerating culture parasitemia by Giemsa-stained thin culture smears and microscopy. Interestingly, we did observe a lower asexual growth rate of *Pfsrpk1^−^* parasites than the WT *Pf*NF54, which was most prominent at the end of the second replication cycle (Fig. 2a). To determine the underlying defect that might cause the lower growth rates, we quantified the number of daughter merozoites per schizont for *Pfsrpk1^−^* in comparison to WT *Pf*NF54 parasites. Strikingly, this revealed that the average number of merozoites per schizont in *Pfsrpk1^−^* was reduced to approximately half in comparison to WT *Pf*NF54 parasites (Fig. 2b and Fig. S1e). To better visualize the number of merozoites in late schizonts, we next performed IFAs using an anti-MSP1 antibody, which marks the merozoite surface, and anti-tubulin X antibody, which marks the subpellicular microtubule network. This further confirmed the reduction in formation of daughter merozoites in *Pfsrpk1^−^* schizonts (Fig. 2c). These data show that the *Pf*SRPK1 is involved in regulating schizogony, and the lack of this kinase leads to the generation of fewer daughter merozoites. This accounts for the observed slower asexual replication rate in the *Pfsrpk1^−^* parasites.

We next analyzed the effects of *PfSRPK1* gene deletion on sexual-stage parasite development. Asexually replicating WT *Pf*NF54 and two clones of *Pfsrpk1^−^* (clones 1B4 and 2C8) parasites were induced to generate gametocytes *in vitro* using standard methods (40). The ability of *Pfsrpk1^−^* to undergo gametocytogenesis was analyzed by Giemsa-stained thin smears over a 14-day *in vitro* culture. This revealed development of various stages of gametocytes (Fig. S1f). Gametocytemia was scored for WT *Pf*NF54 and *Pfsrpk1^−^* on day 15 of *in vitro* culture using Giemsa-stained thin culture smears. While *Pfsrpk1^−^* parasites were able to develop into mature stage V gametocytes, they exhibited a reduced gametocytemia compared to WT *Pf*NF54 parasites (Fig. 2d). We performed IFAs using anti-*Pf*P230p antibody, which marks stage V male gametocytes, and anti-*Pf*g377 antibody, which marks female gametocytes, to better visualize the formation of male and female gametocytes. This showed that *Pfsrpk1^−^* parasites can differentiate into both genders (Fig. 2e).

### *Pfsrpk1^−^* parasites exhibit a complete defect in male gametogenesis and cannot infect the mosquito vector.

**FIG 3:**
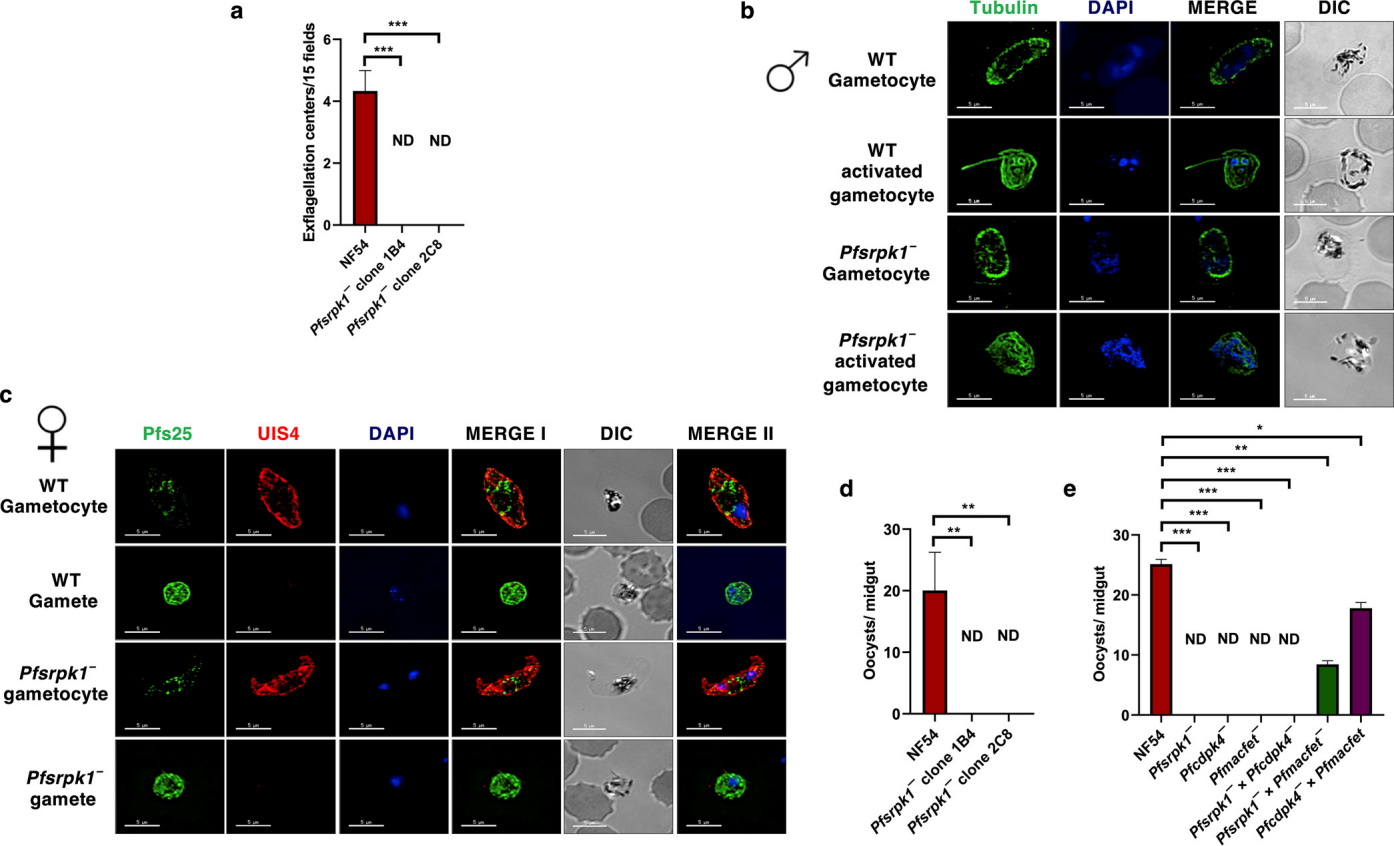
*Pfsrpk1^−^* parasites do not form male gametes and cannot infect the mosquito vector. (a) Number of exflagellation centers (vigorous flagellar beating of emerging microgametes in clusters of RBCs) per field in 15 random fields of view at 15 min postactivation. *Pfsrpk1^−^* (clones 1B4 and 2C8) gametocytes did not show any formation of exflagellation centers. Data were averaged from three biological replicates and are presented as the mean ± standard deviation (SD). (b) The cultures of mature stage V gametocytes were activated for 20 min *in vitro* by addition of human serum and RBCs for WT *Pf*NF54 or *Pfsrpk1^−^* parasites. The activated and nonactivated parasites were stained for α-tubulin (green), a male-specific marker. Note the male gamete emerging from an exflagellating male gametocyte in the WT *Pf*NF54. No emerging microgametes were observed in activated *Pfsrpk1^−^* gametocytes. DIC, differential interference contrast; DAPI, 4′,6-diamidino-2-phenylindole. Symbols for male gametocytes are shown on the left sides of the image panels. (c) The activated and nonactivated parasites were stained for *Pf*UIS4 (red), a marker for parasitophorous vacuole, and anti-*Pf*s25 (green), a marker for female gametes in an IFA. α-*Pf*s25 staining (green) showed female gamete formation for WT *Pf*NF54 and *Pfsrpk1^−^*. (d) WT *Pf*NF54 and *Pfsrpk1^−^* gametocytes were fed to A. stephensi mosquitoes, and the numbers of oocysts per mosquito midgut were enumerated on day 7 postfeed. The plot depicts the number of oocysts per mosquito fed from 3 independent experiments (*n* = 3). Data were averaged from three biological replicates with a minimum of 50 mosquito guts and presented as the mean ± standard deviation (SD). (e) Oocyst formation of WT *Pf*NF54, *Pfsrpk1^−^*, *Pfcdpk4^−^*, *Pfmacfet^−^*, *Pfsrpk1^−^* × *Pfcdpk4^−^*, *Pf srpk1^−^* × *Pfmacfet^−^*, and *Pfcdpk4^−^* × *Pfmacfet^−^*. *In vitro* genetic crosses demonstrated that the *Pfsrpk1^−^* showed productive cross-fertilization with the *Pfmacfet^−^* parasites (which produces functional males only) and not with *Pfcdpk4^−^* (which produces functional females only) (error bar indicates mean ± SD; *n* = 2).

To analyze the ability of *Pfsrpk1^−^* mature gametocytes to undergo gametogenesis, stage V WT *Pf*NF54 and *Pfsrpk1^−^* gametocytes were activated by addition of human serum (type O positive [O^+^]) and a temperature decrease from 37°C to room temperature (RT). Exflagellation centers, each indicating the emergence of microgametes from activated male gametocytes, were measured in 15 random fields of microscopic view. This revealed that *Pfsrpk1^−^* gametocytes did not form any exflagellation centers (Fig. 3a), indicating a complete defect in male gametogenesis. To further analyze the exflagellation defect, IFAs were performed using thin culture smears from WT *Pf*NF54 and *Pfsrpk1^−^* activated gametocytes (20 min postactivation), and parasites were stained with anti-tubulin antibody (for male gametocytes and gametes). To better visualize female gamete egress, IFAs were performed using thin culture smears from WT *Pf*NF54 and *Pfsrpk1^−^* activated gametocytes (20 min postactivation) with anti-PfUIS4 antibody (for parasitophorous vacuole) and with anti-*Pf*s25 antibody (for female gametes). Indeed, no formation of axonemes was observed for male *Pfsrpk1^−^* gametes (Fig. 3b), while female *Pfsrpk1^−^* gametes appeared normal (Fig. 3c). Together, these results indicate *Pf*SRPK1 is critical for male gametogenesis.

A defect in male gametogenesis is expected to impair mosquito infection by the parasite. We thus conducted transmission experiments using mosquito membrane feeding assays with gametocyte cultures to directly test the impact of the *PfSRPK1* gene deletion on parasite transmission. Stage V gametocytes from WT *Pf*NF54 and *Pfsrpk1^−^* parasites were prepared in an infectious blood meal (IBM) by mixing with type O^+^ human serum and fresh red blood cells (RBCs) and then fed to Anopheles stephensi mosquitoes through standard membrane feeders. Mosquitoes were dissected on day 7 post-IBM for enumeration of midgut oocysts using brightfield microscopy. These experiments showed a complete absence of oocysts for *Pfsrpk1^−^*, while WT *Pf*NF54 parasites infected mosquitoes robustly (Fig. 3d). Taken together, these results reveal that *Pf*SRPK1 is critical for parasite transmission to the mosquito vector, and the block is occurring at the level of male gametogenesis.

After determining the role of *Pf*SRPK1 in male gamete formation, we further analyzed the fertility of male and female *Pfsrpk1^−^* gametes. Since gametocytes in *Plasmodium* occur together and it is not possible to separate them, we performed genetic crosses between *Pfsrpk1^−^* parasites and transgenic parasite lines, which either formed fertile female gametes only (*Pfcdpk4^−^*) (8) or fertile male gametes only (*Pfmacfet^−^*) (41). WT *Pf*NF54, *Pfsrpk1^−^*, *Pfcdpk4^−^*, and *Pfmacfet^−^* gametocytes were generated in *in vitro* culture for 15 days, and cultures were first fed individually to female A. stephensi mosquitoes. For crosses, the gametocytes from these parasites were mixed as follows: *Pfsrpk1^−^* × *Pfcdpk4^−^*, *Pfsrpk1^−^* × *Pfmacfet^−^*, *Pfcdpk4^−^* × *Pfmacfet^−^*. Mosquitoes were dissected on day 7 postfeeding to enumerate midgut oocysts for all the feeds. While WT *Pf*NF54 gametocytes infected mosquito midguts robustly, *Pfsrpk1^−^* gametocytes did not (Fig. 3e), nor did the *Pfcdpk4^−^* and *Pfmacfet^−^* parasites, as expected. The *Pfsrpk1^−^* × *Pfcdpk4^−^* cross also showed no transmission. However, in the *Pfsrpk1^−^* × *Pfmacfet^−^* cross, oocysts were observed, albeit in reduced numbers. This indicated productive fertilization of *Pfsrpk1^−^* female gametes by *Pfmacfet^−^* male gametes (Fig. 3e). The reduction of oocyst numbers, however, suggested a reduction in successful fertilization events. Oocyst development was also observed in the *Pfcdpk4^−^* × *Pfmacfet^−^* (positive control) cross. These experiments demonstrate that *Pf*SRPK1 is essential for parasite transmission via a male-specific function.

### *PfSRPK1* deletion leads to widespread perturbation of transcript abundance and dysregulation of splicing.

**FIG 4:**
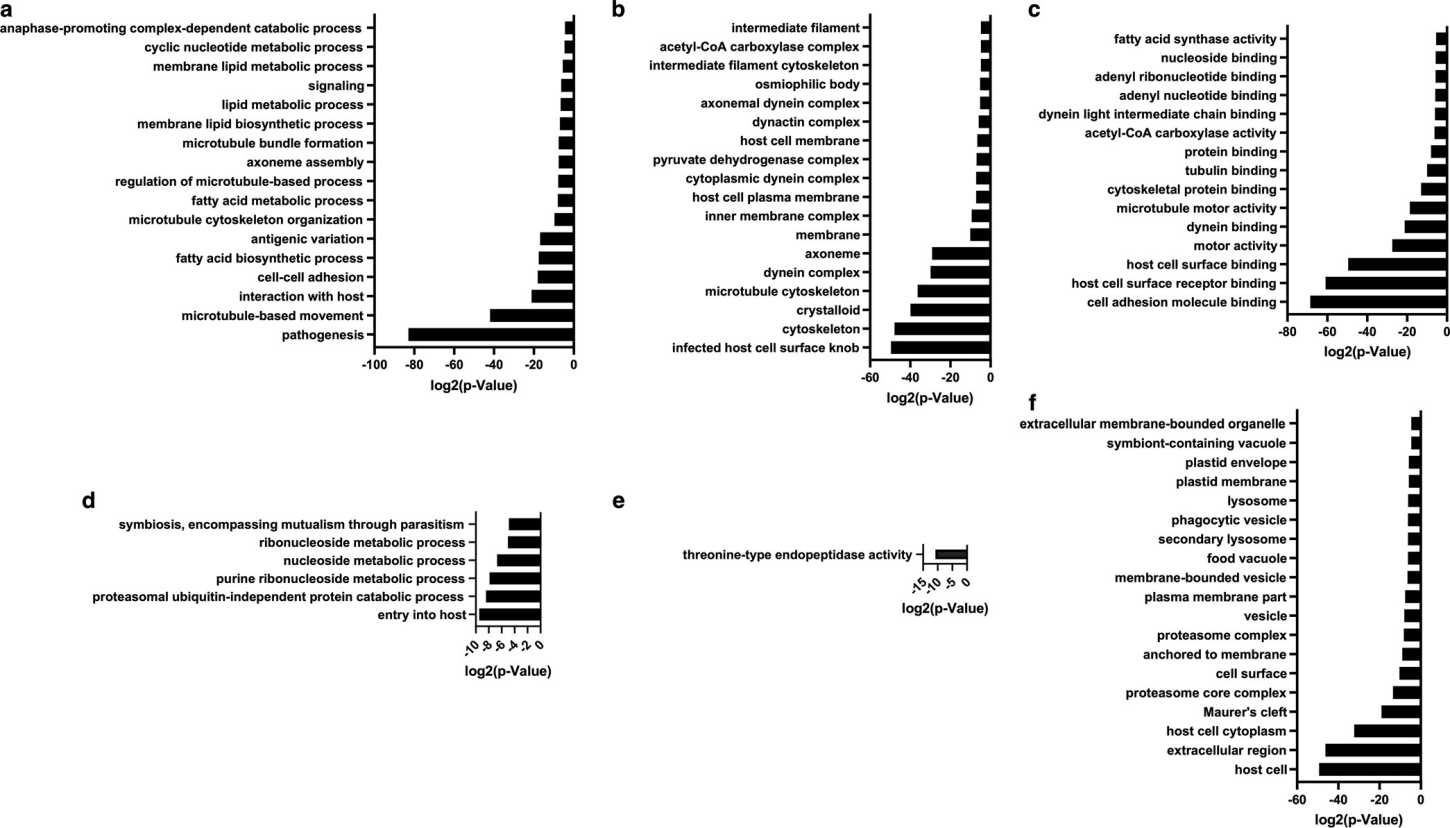
Disruption of *PfSRPK1* results in extensive perturbation of transcript abundance. (a to c) Gene ontology terms for biological processes (a), cellular components (b), and molecular functions (c) of transcripts with reduced abundance are provided and highlight transcript perturbations for proteins involved in key biological processes that are impacted by *Pf*SRPK1 deletion. Log_2_ (*P* values) are indicated on the *x* axes for all the categories. (d to f) Biological processes (d), molecular functions (e), and cellular components (f) of transcripts with increased transcript abundance are provided and highlight transcript perturbations for proteins involved in key biological processes that are impacted by *Pf*SRPK1 deletion. Log_2_(*P* values) are indicated on the *x* axes for all the categories.

Since *Pfsrpk1^−^* gametocytes do not form male gametes, we further investigated the transcriptional changes in *Pfsrpk1^−^* parasites that might explain this severe phenotype. We performed bulk RNA-seq and determined differentially expressed genes (DEGs) between WT *Pf*NF54 and *Pfsrpk1^−^* stage V gametocytes. We chose to analyze these stages, as they did not show any overt defects in the absence of *PfSRPK1* but are the stages from which male gametes form. This analysis revealed 1,202 DEGs, out of which 267 were upregulated and 935 were downregulated in *Pfsrpk1^−^* gametocytes (Data Set S1). To further understand the DEGs dysregulated in *Pfsrpk1^−^*, gene term ontology enrichment analysis was performed, which revealed that transcripts encoding proteins related to microtubule-based movement, microtubule cytoskeletal organization, lipid metabolism, anaphase-promoting complex-dependent catabolic processes, and cell signaling were significantly downregulated (Fig. 4a to c and Data Set S2). On the other hand, transcripts encoding proteins involved in nucleotide metabolism, entry into host (invasion/egress), host cell, cell surface, and Maurer’s clefts were upregulated (Fig. 4d to f and Data Set S2). Interestingly, members of heterochromatin-associated multicopy gene families, including members of the *var*, *rifin*, *PHIST*, and *STEVOR* gene families, were both downregulated and upregulated (Fig. S2 and Data Set S1). Given that these parasite gene families are generally associated with heterochromatin and that SRPK1 in other organisms is known to regulate the chromatin state and thus gene expression (35), it is possible that a disruption of chromatin positioning leads to dysregulation of this group of genes in *Pfsrpk1^−^* parasites.

To explore changes in transcript splicing in *Pfsrpk1^−^*, RNA-seq data were analyzed for differentially spliced genes in *Pfsrpk1^−^* compared to WT *Pf*NF54. For this, differential exon usage (DEU) was calculated using DEXSeq as evidence for changes in splicing. DEXSeq is a Bioconductor package which employs generalized linear models and identifies changes in the expression of exons that are not simply the outcome of overall up- or downregulation of the transcripts (42). This analysis revealed differential exon usage for 260 parasite transcripts, indicating significant changes in splicing patterns in *Pfsrpk1^−^* stage V gametocytes (Fig. S3 and Data Set S3). Interestingly, among these differentially spliced transcripts, 129 were also downregulated, while 6 were upregulated in *Pfsrpk1^−^* stage V gametocytes (Data Set S1).

To further analyze the functional significance of affected transcripts in *Pfsrpk1^−^*, gene term ontology enrichment analysis was performed, which revealed that transcripts encoding proteins related to axoneme, dynein complex, microtubule-associated complex, cytoskeletal organization, and cell adhesion were significantly affected (Fig. S3a to c). Importantly, this analysis also revealed that splicing of transcripts which are not DEGs was also affected. Since SRPKs exert their role in splicing via phosphorylation of SR proteins, incorrect splicing might be one factor that leads to increased transcript degradation and thus might explain reduced transcript abundance for DEGs to some degree in *Pfsrpk1^−^*.

### *Pfsrpk1^−^* parasites show altered transcript abundance for proteins involved in microtubule formation, signaling, and lipid metabolism.

**FIG 5:**
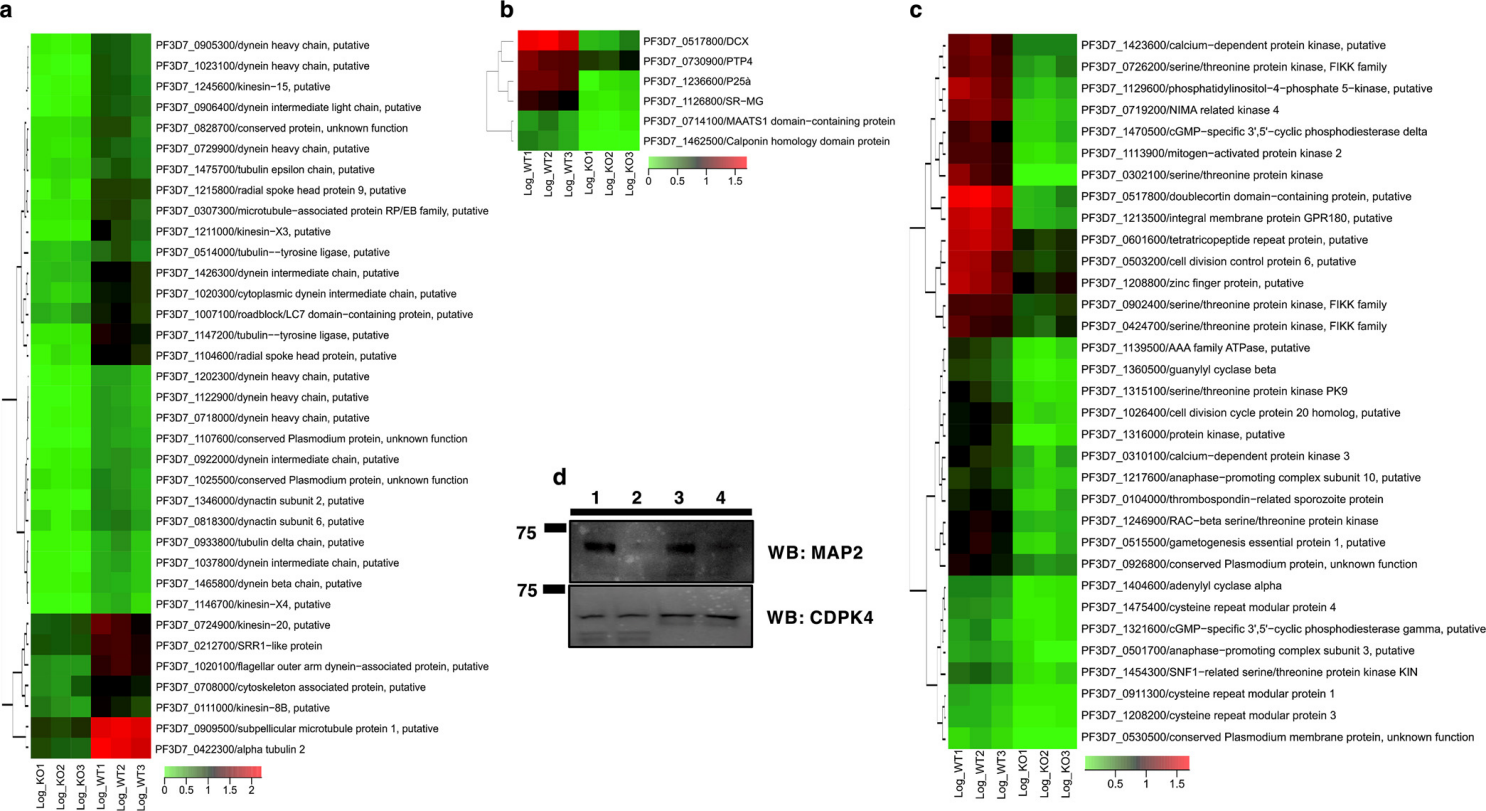
Deletion of *PfSRPK1* results in perturbation of transcript abundance for genes encoding proteins involved in microtubule/cilium formation and signaling. (a) Heatmaps showing differentially expressed genes (DEGs) encoding microtubule/cilium formation proteins that are downregulated in *Pfsrpk1^−^* gametocytes. (b) Heatmaps showing DEGs encoding gametocytogenesis-related genes that are downregulated in *Pfsrpk1^−^* gametocytes. Scale bar indicates log_2_ fold change of transcript-per-million (TPM) values of the samples in expression. (c) Heatmaps showing that DEGs encoding proteins involved in cell signaling and cyclic nucleotide metabolism are downregulated in *Pfsrpk1^−^* gametocytes. Scale bar indicates log_2_ fold change of TPM values of the samples in expression. (d) Western blot analysis of *Pf*MAP2 in WT *Pf*NF54 and *Pfsrpk1^−^* gametocytes, showing reduced protein abundance for *Pf*MAP2 in *Pfsrpk1^−^* gametocytes. *Pf*CDPK4 abundance is shown as the loading control. 1, 2, 3, and 4 represent lysates prepared from two independent experiments. 1 and 3, WT *Pf*NF54; 2 and 4, *Pfsrpk1^−^* gametocytes.

Gene ontology (GO) analysis of DEGs revealed that several transcripts that encode proteins involved in microtubule-based processes and cytoskeletal organization exhibited reduced abundance. These genes comprised those coding for several radial spoke head proteins, dynein light/heavy chain proteins, tubulin proteins, tubulin tyrosine ligase proteins, and kinesins, which can be relevant to axoneme formation during microgametogenesis (Fig. 5a and Data Set S2).

Recent studies have implicated parasite kinesins in spindle assembly, axoneme formation, and cellular morphology (43). Therefore, downregulation of these proteins fits well with the axoneme formation defect that we observed in the *Pfsrpk1^−^* males. Interestingly, some of the genes identified in a previous transposon mutagenesis study (44), such as MAATS1 domain-containing protein (PF3D7_0714100), calponin homology domain protein (PF3D7_1462500), PTP4 (EMP1-trafficking protein, PF3D7_0730900), and P25α family protein (PF3D7_1236600), were downregulated in *Pfsrpk1^−^* (Fig. 5b and Data Set S1). In addition, *Pf*apicortin-DCX (PF3D7_0517800) was also downregulated (Fig. 5b and Data Set S1).

We also found that several transcripts encoding signaling proteins, such as kinases (MAP2, CDPK3, PKB, phosphatidylinositol-4-phosphate 5-kinase [PI4P5K], FIKK9.5, FIKK97.1, NEK2, NEK3, and NEK4) and cyclic nucleotide signaling metabolism-related genes (ACα, GCβ, PDEγ, and PDEδ), were downregulated in *Pfsrpk1^−^* gametocytes (Fig. 5c and Data Set S1). This suggests that *Pf*SRPK1 deletion affects cellular signaling events, which is relevant to gametogenesis. *Pf*MAP2 kinase is known to regulate axonemal beating and male gametogenesis (13). Using anti-*Pf*MAP2 antibodies, we observed that *Pf*MAP2 is coexpressed with *Pf*SRPK1 in gametocytes (Fig. S4a). Furthermore, Western blot analysis showed that *Pf*MAP2 protein expression levels were indeed reduced in *Pfsrpk1^−^* gametocytes (Fig. 5d). In addition, transcripts for gametogenesis essential protein 1 (GEP1) and gamete egress protein (GEP), which are known to play a role in gametogenesis in rodent malaria parasites (11, 45), were also downregulated (Data Set S1).

Another category of downregulated DEGs was related to fatty acid biosynthesis and lipid metabolism (Fig. 4a to c and Fig. S4b). Fatty acid composition changes during gametocytogenesis and is known to regulate this process in P. falciparum (46, 47). Also, lipid composition is different between male and female gametocytes (48). This lipid dimorphism precedes gametocyte activation and possibly reflects sex-specific functions (48). One of these genes, elongation of fatty acids protein (ELO3), which is known to be involved in gametocytogenesis (44), was among the downregulated DEGs (Fig. S4b and Data Set S1). Since phospholipids are the main component of membranes and an activated male gametocyte forms eight flagellated microgametes, various phospholipids are likely required for the rapid membrane generation. Downregulation of several DEGs encoding lipid metabolism might, in part, explain the reduced ability of *Pfsrpk1^−^* to undergo male gametogenesis.

### *Pfsrpk1^−^* parasites show altered transcript abundance for genes encoding components of the inner membrane complex, egressome, osmophilic bodies, and crystalloids.

**FIG 6:**
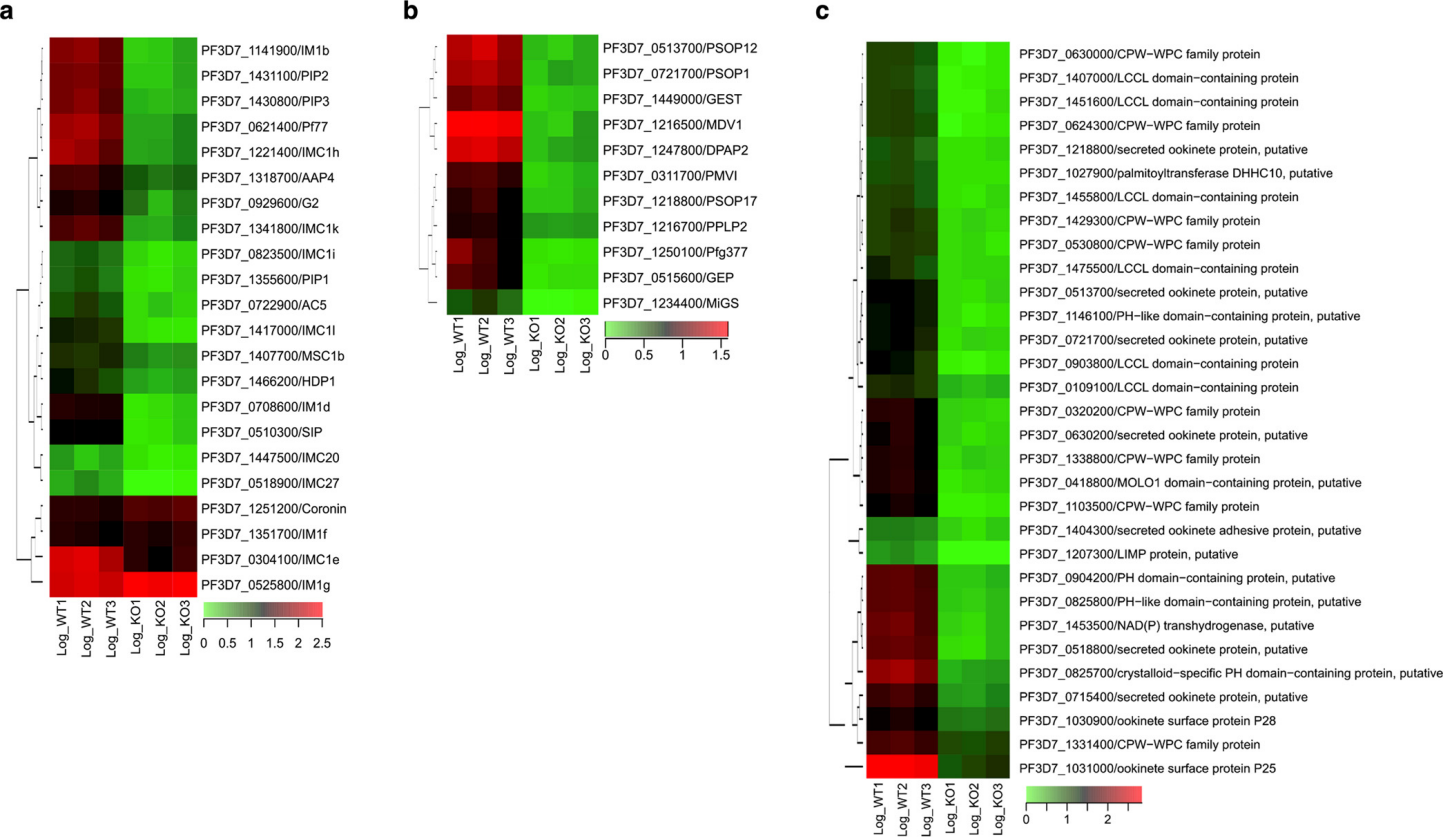
Disruption of *PfSRPK1* results in perturbation of transcript abundance for genes encoding constituent proteins of the inner membrane complex (IMC), osmophilic bodies, and crystalloid. (a) Heatmaps showing DEG transcripts for IMC components that are downregulated in *Pfsrpk1^−^* gametocytes. (b) Heatmaps showing DEG transcripts encoding osmophilic body components that are downregulated in *Pfsrpk1^−^* gametocytes. Scale bar indicates log_2_ fold change in expression. (c) Heatmaps showing that DEG transcripts encoding ookinete/crystalloid components are downregulated in *Pfsrpk1^−^* gametocytes. Scale bar indicates log_2_ fold change of TPM values of the samples in expression.

The gene ontology analysis retrieved annotations for numerous DEGs encoding proteins of the inner membrane complex (IMC), gametocyte osmophilic bodies, and crystalloids, the latter of which are special organelles present in early mosquito stages postfertilization (Fig. 4b, Fig. 6a to c, and Data Sets S1 and S2). The IMC is a membrane system that undergirds the parasite plasma membrane and is, in turn, supported by microtubules. Previous studies have shown the IMC-regulated cellular deformability of gametocytes (49) and downregulation of IMC components lead to a block in sexual-stage development (50). In addition, we observed downregulation of the transcript encoding homeodomain protein 1 (HDP1) in *Pfsrpk1^−^* (Fig. 6a and Data Set S1). *Pf*HDP1 is involved in regulating gametocytogenesis via expansion of the IMC (51).

During the egress of gametocytes from the infected RBCs, several egress molecules are released by exocytosis. In the rodent malaria parasite Plasmodium berghei, these molecules have been collectively described as components of a so-called “egressome” (52, 53). Transcripts of several P. falciparum orthologs of these egress-related proteins, such as *Pf*g377, PSOP1, PSOP12, PSOP17, male development gene 1 (MDV-1), GEP, DPAP2, gamete egress and sporozoite traversal protein (GEST), microgamete surface protein (MiGS), plasmepsin VI (PMVI), and perforin-like protein (PPLP2), were downregulated in *Pfsrpk1^−^* gametocytes (Fig. 6b and Data Set S1). Most of these proteins are also known components of vesicles called osmiophilic bodies (OBs), which have essential functions during gametocyte egress (54–56). In P. falciparum, OBs are predominantly studied in female gametocytes (57), while in the rodent malaria parasites P. berghei and P. yoelii, OBs are described in both female and male gametocytes, with the male OBs being smaller in size and club-shaped (54, 55). Downregulation of PPLP2, which is critical for gametogenesis (58) in *Pfsrpk1^−^* gametocytes (Fig. 6b), further supports the sexual-stage defects in *Pfsrpk1^−^*.

Interestingly, transcripts encoding P. falciparum orthologs of the P. berghei crystalloid proteins, such as LCCL/lectin adhesive-like protein/CCp (LAP) family proteins LAP1, LAP2, LAP3, LAP4, LAP5, and LP6; CPW-WPC family proteins, putative secreted ookinete protein 1 (PSOP1), PSOP6, PSOP12, PSOP17, and PSOP20; NAD(P) transhydrogenase (NTH); PH-like domain-containing proteins; and *Pf*25 and *Pf*28 were also downregulated in *Pfsrpk1^−^* (Fig. 6c and Data Set S1).

## DISCUSSION

**FIG 7:**
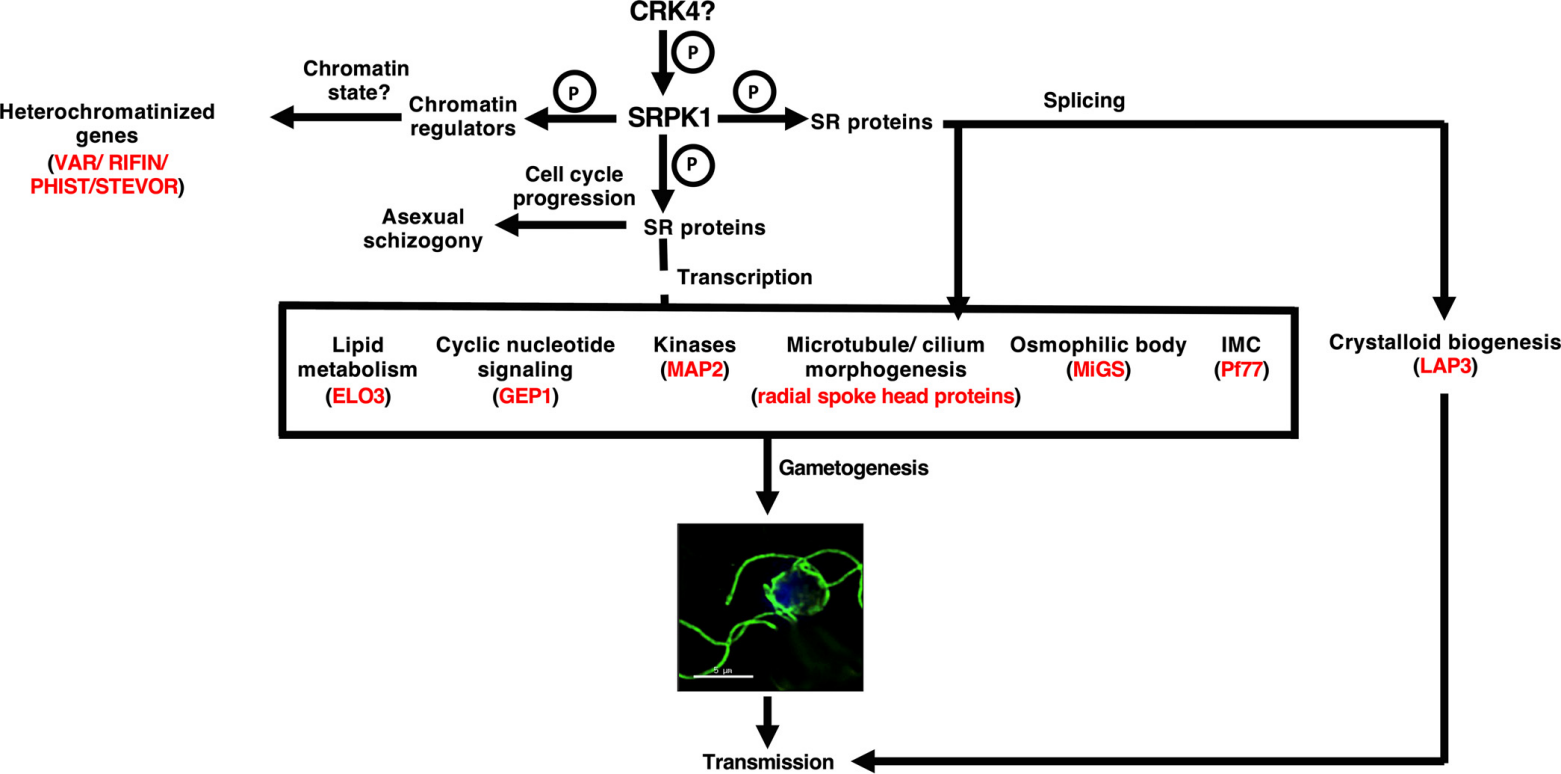
Model of the links between transcriptome perturbations in *PfSRPK1*-deficient parasites, (a)sexual-stage phenotype, and *Pf*SRPK1 function. Possible upstream kinases *Pf*CRK4-mediated phosphorylation likely regulate *Pf*SRPK1 activity. *Pf*SRPK1 phosphorylates SR proteins, which regulate mRNA splicing and thereby control expression of proteins involved in microgametogenesis and also splicing of mRNAs destined for storage and translation postfertilization. *Pf*SRPK1 might also be involved in controlling gene expression by regulating the chromatin state and cell cycle by phosphorylating chromatin regulators. Annotated and predicted protein function was based on gene ontology terms and manual curation. An example of a protein in each category is given (red). Letter P inside circle denotes phosphorylation.

Malaria parasites replicate asexually within the infected red blood cells, with each round of infection culminating in the formation and release of daughter merozoites, which infect new cells and form the next generation of asexual stages. A small subset of infected cells produces merozoites that are committed to developing into sexual-stage gametocytes. These gametocytes are taken up by the mosquito vector in which fertilization occurs, and the sexual phase of the life cycle is completed. Previous studies have suggested *Pf*SRPK1 to be an essential kinase for asexual replication, as it was reported to be refractory to gene disruption (37). Using CRISPR/Cas9, we succeeded in deleting *PfSRPK1* and showed that *Pf*SRPK1 is not essential but plays an important role in asexual parasite replication. In addition, *PfSRPK1*did have an essential role in male microgametogenesis. We further showed that *Pf*SRPK1 deletion had a pronounced impact on the stage V gametocyte transcriptome, and closer analysis of the observed perturbations enabled us to develop a working model for *Pf*SRPK1 function (Fig. 7).

SRPKs are key regulators of splicing and alternate splicing of transcripts in eukaryotes. SRPKs exert this function by phosphorylating SR proteins, which also regulates their nucleocytoplasmic shuttling (34). SRPKs further influence additional steps of RNA maturation and also regulate various cellular processes such as chromatin organization, cell cycle progression, and metabolic signaling (59). Previous studies have shown that *Pf*SRPK1 possesses all the 11 kinase subdomains and that recombinantly expressed *Pf*SRPK1 shows kinase activity and can phosphorylate recombinantly expressed *Pf*SR1 (60). We found that *Pf*SRPK1 was expressed throughout asexual intraerythrocytic development and in gametocytes. The localization appeared to be within the cytoplasm of these parasite stages. Interestingly, *Pf*SRPK1 was absent in the female stage V gametocytes, which indicated a role in male gametocytes only. We demonstrated that *Pfsrpk1^−^* parasites formed only about half the number of daughter merozoites within each schizont, significantly reducing asexual parasite replication and parasitemia increase in culture, compared to WT *Pf*NF54 parasites. It is possible that *Pf*SRPK1 functions downstream of certain key cell cycle regulators such as cdc2-related protein kinase 4 (*Pf*CRK4), which has been shown to play a role in DNA replication during intraerythrocytic schizogony (61). In fact, *Pf*SRPK1 is heavily phosphorylated with numerous phospho-sites (Y^29^, T^30^, S^32^, S^293^, S^355^, S^400^, S^503^, S^505^, S^508^, T^511^, S^566^, T^569^, S^576^, S^601^, S^646^, T^649^, S^880^, S^882^, S^892^, S^893^, S^969^, S^1040^, T^1201^, S^1248^, S^1248^, S^1279^, and S^1280^) (PlasmoDB). Importantly, it was observed to be hypophosphorylated upon *Pf*CRK4 knockdown at amino acids S^355^, S^601^, S^880^, S^893^, S^969^, S^1040^, S^1248^, and S^1279^ (61), suggesting that *Pf*CRK4 functions upstream of *Pf*SRPK1 in schizont stages. Furthermore, a reduced growth rate caused by a decrease in the average number of daughter merozoites per schizont has been reported for other *Plasmodium* protein kinases, *Pf*CRK5 (62), *Pf*PK7 (63), and *Pf*CDPK7 (64). Thus, it will be of interest to explore the interplay between these kinases and *Pf*SRPK1 in governing the control of daughter merozoite formation in the future. In the rodent malaria parasite P. berghei, SRPK1 has a minor function during asexual blood-stage development, and the peak replication rate during exponential parasite growth is not reduced (65). Our observations with *Pfsrpk1^−^* parasites are in agreement with previous studies performed using pharmacological inhibitors, showing *Pf*SRPK1 to be important for asexual blood-stage development (38). In contrast, a recent study reported that clinical field isolates from the Gambia with premature stop codons in *PfSRPK1* within the spacer region present between the kinase domains (58-253 and 681-850) outcompeted WT parasites in AlbuMAX-supplemented culture medium (66). The authors concluded that the better growth rate shown by the *SRPK1* mutant with premature stop codon may indicate that disruption is adaptive in certain conditions of initial *in vitro* culture establishment (66).

We further found that *Pfsrpk1^−^* parasites differentiate into gametocytes and formed mature stage V gametocytes, albeit at lower gametocytemia than the WT NF54 parasites. Likely, the reduced gametocytemia was a direct consequence of the reduced number of merozoites originating from *Pfsrpk1^−^* schizonts, which would also result in a reduction of merozoites committing to gametocytogenesis. Strikingly, we demonstrated that *Pf*SRPK1 has a critical role during microgametogenesis, as male *Pfsrpk1^−^* gametocytes did not form microgametes. In contrast, we observed no discernible defect in *Pfsrpk1^−^* macrogamete formation. Furthermore, we showed that *Pfsrpk1^−^* parasites did not transmit to the mosquito vector. Utilizing genetic crosses with transgenic parasite lines producing either fertile female (8) or male gametes(41), we showed that *Pf*SRPK1 has mainly a male gender function. Female *Pfsrpk1^−^* gametes were fertile, but our observation that their transmission in the cross with the female-deficient line was reduced might indicate some function in females. On the other hand, we did not observe *Pf*SRPK1 expression in female stage V gametocytes. Thus, this requires further investigation.

In eukaryotic cells, alternative splicing has the potential to increase and diversify the proteome size from a limited set of genes (67, 68). SRPKs are key regulators of splicing and alternative splicing of transcripts. Although SRPKs are present in both the cytoplasm and nuclear compartments, their function in the cytoplasm is best explored. They regulate splicing via phosphorylation of the (arginine/serine-rich) RS domain of SR protein-splicing factors, which is required for their nuclear translocation. *Pf*SRPK1 has been suggested to interact with *Pf*SR1 and phosphorylates it *in vitro* (60). Previous studies have shown that critical catalytic residue (K89) is pivotal for kinase (catalytic) activity of *Pf*SRPK1, and recombinantly expressed catalytically active *Pf*SRPK1 is known to influence pre-mRNA splicing *in vitro* (60). Interestingly, expression of a splicing factor, *PfS*R-MG (PF3D7_1126800), was downregulated and was the only splicing factor to be downregulated in *Pfsrpk1^−^* (see Data Set S1 in the supplemental material). SR-MG is involved in male gametogenesis in P. berghei (69), and *Pf*SR-MG is dispensable for asexual blood-stage replication (70) and shows high expression in gametocytes (PlasmoDB). Thus, to gain insight into potential transcriptome perturbations caused by *PfSRPK1* deletion, we performed bulk RNA-seq on stage V gametocytes of WT *Pf*NF54 and *Pfsrpk1^−^* parasites. We chose to analyze this parasite stage, as it precedes gametogenesis but did not yet present with an overt phenotype. RNA-seq followed by differential exon usage analysis revealed significant alterations in splicing of transcripts in *Pfsrpk1^−^* parasites. This fits well with the predicted role for SRPK1 in regulation of SRs and splicing in the parasite. Comparative RNA-seq analysis of WT *Pf*NF54 and *Pfsrpk1^−^* stage V gametocytes further revealed widespread changes in transcript abundance with 1,202 DEGs, out of which 267 were upregulated and 935 were downregulated in *Pfsrpk1^−^*gametocytes. The incorrect splicing might also lead to increased transcript degradation. Indeed, the abundance of 129 out of 260 transcripts that showed altered splicing was reduced in *Pfsrpk1^−^*.

Gene ontology analysis showed that DEGs with reduced transcript abundance encoded microtubule/cilium morphogenesis cell motility-related proteins such as tubulin chains, tubulin binding apicortin subunits (P25α and DCX), dynein chains (light, intermediate, and heavy), kinesins, and radial spoke head proteins, which are all relevant for flagellum formation and function. Apicortin is a microtubule binding protein which consists of two domains, a partial P25α and doublecortin (DCX) domain (71). These domains are uniquely present in distinct microtubule-interacting proteins in various cell types of vertebrates and *Plasmodium* (72–74) but present in the same protein of the related apicomplexan parasite *Toxoplasma* (75). P. falciparum apicortin also displays tubulin binding properties (74). These results might explain the observed defect in *Pfsrpk1^−^* microgamete formation.

Interestingly, several genes which encode proteins present in osmophilic bodies were also downregulated. Osmophilic bodies have a role in gametogenesis (54–57). One of these proteins, PPLP2, has been shown to play a critical role during gametogenesis in P. falciparum (58). Interestingly, transcripts encoding several signaling proteins, such as MAP2 kinase; NEK2, -3, and -4 kinase; PKB; CDPK3; PI4P5K; and cyclic nucleotide-signaling metabolism-related proteins (ACα, GCβ, PDEγ, and PDEδ), were depleted in *Pfsrpk1^−^* gametocytes. *Pf*MAP2 kinase has been shown to be critical for male gametogenesis (13), and we showed that *Pf*MAP2 protein levels are reduced in *Pfsrpk1^−^* gametocytes. This would contribute to the exflagellation defect in *Pfsrpk1^−^* gametocytes. Given that several parasite transcripts encoding kinases are downregulated in *Pfsrpk1^−^* gametocytes, *Pf*SRPK1 appears to be a central kinase directly or indirectly regulating components of various signaling cascades leading to male gametogenesis.

Interestingly, another group of DEGs involved those encoding IMC proteins. While the IMC has a central role in motility in invasive stages such as merozoites, ookinetes, and sporozoites, IMC proteins are expressed throughout the parasite cycle stages (49, 76, 77). They are involved in enhanced cellular deformability of gametocytes (49) and gametocyte development (50). Furthermore, activation of gametocytes is a very rapid process (54) and is dependent on the disassembly of the IMC. The dissolved IMC vesicles are hypothesized to form “nanotubes,” which are membranous cell-to-cell connections (78) and can facilitate mating of the male and female gametes (78). Reduced transcript abundance for several DEGs encoding IMC components might thus cause reduced gametogenesis and/or fertility in *Pfsrpk1^−^* parasites. These hypotheses would require further investigation.

Surprisingly, another group of DEGs with reduced transcript abundance in *Pfsrpk1^−^* parasites were genes encoding proteins of the crystalloid. These proteins are expressed in parasite ookinetes and early oocyst stages and contained in specialized organelles called crystalloids, which appear as clusters of tightly packed small spherical units in electron microscopy and are important for regulating sporogony (79, 80). Several of these transcripts are translationally repressed by the development of zygote-inhibited (DOZI) mRNA storage complex in P. berghei (20), while some of the mRNAs are regulated by Puf protein family protein Puf2 in P. falciparum (81). Recent studies in rodent malaria parasites have identified components of crystalloid proteins with LCCL lectin adhesive proteins (LAPs), CPW-WPC family proteins, secreted ookinete proteins (SOPs), and several PH domain-containing proteins (82). We found that most transcripts for P. falciparum orthologs of these crystalloid-specific proteins are downregulated in *Pfsrpk1^−^* parasites, along with transcripts for RNA binding proteins of the Puf family, Puf1 and Puf2. Puf1 and Puf2 are expressed in gametocytes and are known to regulate sexual development, sex differentiation, and maintenance of gametocytes (83, 84). RNA immunoprecipitation of Puf2-green fluorescent protein (GFP) parasites revealed previously that NTH, plasmepsin VI, a conserved *Plasmodium* protein (PF3D7_1248400), *Pf*25, and *Pf*28 are bound by Puf2 (81). These results indicate that *Pf*SRPK1 might be regulating the expression of mRNAs that are stored and repressed in stage V gametocytes in preparation for postfertilization events in the mosquito vector through yet-uncharacterized mechanisms.

We also observed significant changes (both increased and decreased) in the transcript abundance of members belonging to multigene heterochromatin-associated families such as P. falciparum *VAR*, *RIFIN*, *STEVOR*, and *PHIST* in the *Pfsrpk1^−^* parasites. These gene families were reported to be expressed in gametocytes (85–89). It has been suggested that *VAR* gene expression may continue to provide variable antigenic expression in gametocytes during maturation inside the human host (85). Similarly, *PHIST* family proteins are exported during gametocytogenesis (86) and control infected RBC rigidity (90). Host cell deformability and rigidity changes occur late in gametocyte development and are possible crucial factors in gametocyte transmission to the mosquito vector (91). In fact, an EMP1-trafficking protein, PTP4 (90), which also has a role in gametocytogenesis (44), was also downregulated in *Pfsrpk1^−^*. Given the role of SRPK1 in regulating mRNA maturation and chromatin accessibility in mammals (35, 92, 93), it is possible that the chromatin landscape is perturbed in *Pfsrpk1^−^* parasites, which might lead to dysregulation of heterochromatinized genes and other genes in the stage V gametocytes. It is also possible that these changes may arise due to allelic exclusion between different clones in the parasite population. These hypotheses would require further investigation.

The work we describe here highlights the role of *Pf*SRPK1 in asexual parasite replication, pre-mRNA splicing, microgametogenesis, male fertility, and transmission to the mosquito vector. Our results also complement earlier studies performed using pharmacological inhibitors (38) and highlight the importance of SRPK1 in asexual blood stages in addition to its role in sexual stages. We establish the role of SRPK1 in pre-mRNA splicing and show that the altered splicing leads to downregulation of male gametogenesis-related genes. We provide compelling evidence that *Pf*SRPK1 regulates both transcript and protein levels of a key gametogenesis-related kinase, *Pf*MAP2. Since SRPK proteins in other organisms are known to regulate chromatin states (35), future studies would focus on understanding *Pf*SRPK1-mediated phosphorylation-dependent chromatin regulation and other parasite processes, which may shed light on molecular mechanisms regulating gametogenesis and parasite transmission to the mosquito vector.

## MATERIALS AND METHODS

### Reagents and primary antibodies.

All the molecular biology reagents were from MilliporeSigma (USA) until otherwise stated. All the oligonucleotides were purchased from Integrated DNA Technologies. The following primary antibodies and antisera and dilutions were used: mouse anti-tubulin antibody (1:250; Sigma-Aldrich; catalog no. T5168), rabbit anti-*Pf*g377 (1:250; kindly gifted by Pietro Alano at Istituto Superiore di Sanità, Italy), and mouse anti-*Pf*P230p (1:200, kindly gifted by Kim C. Williamson, Uniformed Services University of the Health Sciences, USA) (94). The rabbit anti-*Pf*UIS4 (1:100) is described elsewhere (95). The generation of polyclonal mouse antisera against *Pf*SRPK1 (1:50) is described below. Alexa Fluor-conjugated secondary antibodies were purchased from Thermo Scientific.

### P. falciparum culture and transfection.

WT P. falciparum NF54 and *Pfsrpk1^−^* parasites were cultured as asexual blood stages according to standard procedures and received complete RPMI 1640 media supplemented either with 0.5% AlbuMAX II (Thermo Scientific) medium or 10% (vol/vol) human serum changes every 24 h. Gametocytes were generated using O^+^ human RBCs (Valley Biomedical, VA, USA) and O^+^ human serum (Interstate Blood Bank, TN, USA) using a previously published protocol (40). Gametocyte cultures were set up in 6-well plates with a final volume of 5 mL at 1% initial parasitemia and 4% hematocrit. All the cultures were kept at 37°C inside an incubator and supplemented with “malaria gas” containing 5% O_2_, 5% CO_2_, and 90% N_2_.

Oligonucleotide primers used for the creation and analysis of P. falciparum *PfSRPK1^−^* parasites are mentioned in Table S1 in the supplemental material. Deletion of *PfSRPK1* was achieved using CRISPR/Cas9 strategy with double-crossover homologous recombination. Homology regions of *PfSRPK1* upstream (5′) and downstream (3′) of the open reading frame were ligated into plasmid pFCL3 (generated in the lab by modification of the pYC plasmid), as was the 20-nucleotide guide RNA sequence, resulting in the creation of plasmid pFCL3_SRPK1_KO 1. Similarly, pFCL3_SRPK1_KO 2 was generated by cloning a different guide RNA sequence with the same homology arms as pFCL3_SRPK1_KO 1. One hundred micrograms each of these two plasmids was mixed and transfected into the *Pf*NF54 strain using Bio-Rad electroporator following standard methods and selected using 8 nM WR99210 (kindly gifted by Jacobus Pharmaceuticals). Gene deletion was confirmed by a set of genotyping PCRs (Fig. S1). Two individual clones for *Pfsrpk1^−^* (clones 1B4 and 2C8) were used for phenotypic analysis.

### Generation of antisera.

The peptide corresponding to amino acids 1312 to 1335 from the C terminus of *Pf*SRPK1 was conjugated to carrier protein keyhole limpet hemocyanin (KLH), and LIENRDDQNVNKINCKVINKKNSC-KLH was synthesized by Biomatik (Ontario, Canada) and was used for immunization of mice following standard procedures.

### Measurement of asexual blood-stage growth and gametocyte development.

To compare asexual blood-stage replication and growth between the WT *Pf*NF54 and *Pfsrpk1^−^* parasites, parasites were synchronized, and cultures were initiated at an initial parasitemia of 1% at ring stages in 6-well plates and maintained as described above. Parasites were removed at 48 and 96 h for preparation of Giemsa-stained thin culture smears, and parasitemia was scored per 1,000 erythrocytes. To compare gametocyte formation between WT *Pf*NF54 and *Pfsrpk1^−^*, gametocytes were cultured as described above. Parasites were removed on day 15 of *in vitro* culture for preparation of Giemsa-stained blood smears, and gametocytemia was scored per 1,000 erythrocytes.

### Exflagellation, standard membrane feeding assay (SMFA), and oocyst measurements.

For assaying comparative exflagellation, equal volumes of gametocytes from WT *Pf*NF54 and *Pfsrpk1^−^* were mixed with human serum and O^+^ RBCs (50:50) % and incubated at room temperature for 10 min. Exflagellation was scored for WT *Pf*NF54 and P. falciparum *srpk1^−^* parasites via light microscopy by counting exflagellation centers in 10 optical fields of view at ×40 magnification.

For SMFA, infectious blood meal was prepared by mixing stage V gametocytes for WT *Pf*NF54 or *Pfsrpk1^−^* with human serum and O^+^ RBC mixture (50:50) % to achieve a final gametocytemia of 0.5% and loaded on standard mosquito feeders with parafilm attached to their bottom. Mosquitoes were allowed to feed on the blood meal for approximately 25 min, after which feeders were removed, unfed mosquitoes were aspirated out, and mosquito cages were moved to incubators. On day 7 post-blood meal, mosquitoes were dissected for midguts, and oocysts were enumerated under bright-field microscope at ×10 magnification.

### IFAs.

For performing IFAs, smears were prepared on Teflon-coated slides and fixed with 4% paraformaldehyde-0.0025% glutaraldehyde solution for 30 min. Slides were kept in a humidity chamber for each step. Fixed parasites were washed twice with phosphate-buffered saline (PBS) followed by permeabilization with 0.1% Triton X-100-PBS solution for 10 min. Parasites were washed with PBS and blocked with 3% bovine serum albumin (BSA)-PBS for 45 min at RT. Primary antisera prepared in 3% BSA-PBS was added to the parasites, and slides were incubated at 4°C. Antigens were visualized using anti-species secondary antibodies conjugated to Alexa Fluor. Images were obtained using a 100× 1.4-numerical-aperture (NA) objective 90° (Olympus) on a Delta Vision Elite high-resolution microscope (GE Healthcare Life Sciences).

### Comparative RNA-seq and data analysis.

On day 15 of gametocyte development, stage V gametocytes were harvested using saponin lysis. Total RNA from saponin-lysed parasites was extracted using TRIzol (Invitrogen) and Qiagen RNA extraction kit. Following RNA isolation, total RNA integrity was checked using a 2100 Bioanalyzer (Agilent Technologies, Santa Clara, CA). RNA concentrations were measured using the NanoDrop system (Thermo Fisher Scientific, Inc., Waltham, MA). rRNA was removed from total RNA using Illumina Ribo Zero Gold for human, mouse, and rat kit. The libraries were multiplexed and clustered on one lane of a flow cell and loaded on an Illumina HiSeq platform according to the manufacturer’s instructions. After we investigated the quality of the raw data, sequence reads were trimmed to remove possible adapter sequences and nucleotides with poor quality by using Trimmomatic v.0.36. The trimmed reads were mapped to the Plasmodium falciparum reference genome using the STAR aligner v.2.5.2b. BAM files were generated because of this step. Unique gene hit counts were calculated by using featureCounts from the Subread package v.1.5.2. R software v.3.4.1 was used when executing DESeq2 analysis for DEG identification and graphic tools, whereas R v.3.2.5 was used to identify splicing variants using DEXSeq package. All the analyses were performed with default parameters; DEGs were defined as genes with absolute log_2_ fold change (FC) of >1 and an adjusted *P* value of <0.05.

Gene ontology term enrichment analyses were carried out by Cytoscape v.3.9.0 (96) with the Bingo plugin (97). GO terms for P. falciparum genes were downloaded from the PlasmoDB database. GO terms from all three categories were fetched from this and used as input against all the known GO terms in the Bingo plugin. A hypergeometric distribution test was performed at *P* value of ≤0.05 with Bonferroni correction. The network of enriched GO terms thus obtained was reported as the result.

### Statistical analysis.

All data are expressed as mean ± standard deviation (SD). Statistical differences were determined using one-way analysis of variance (ANOVA) with *post hoc* Bonferroni multiple-comparison test or unpaired two-tailed Student's *t* test as indicated. *P* values of <0.05 were considered statistically significant. Significances were calculated using GraphPad Prism 8 and are represented in the figures as follows: ns, not significant, *P* > 0.05; *, *P* < 0.05; **, *P* < 0.01; and ***, *P* < 0.001.

### Ethical statement.

The animal experiments, which involved antisera generation in mice, were performed at the Center for Global Infectious Disease Research (CGIDR), Seattle Children’s Research Institute (SCRI), and prescribed guidelines were followed.

### Data availability.

We confirm that the data supporting the findings of this study are available within the article and/or its supplemental material. The raw fastq files for the RNA-seq data were submitted to NCBI GEO under accession no. GSE212142.
