# Supplementary material for: PfSRPK1 Regulates Asexual Blood Stage Schizogony and Is Essential for Male Gamete Formation
Source: Microbiol Spectr. 2022 Sep 12;10(5):e02141-22. doi: 10.1128/spectrum.02141-22 (PMC9602455; doi:10.1128/spectrum.02141-22)
Supplement: Supplemental file 5 — Fig. S1-S4 and Table S1. Download spectrum.02141-22-s0005.pdf, PDF file, 0.4 MB [file spectrum.02141-22-s0005.pdf]

Supplemental Material include:

Supplementary Data S1. Summary of DEGs between WT NF54 and *Pfsrpk1*$^{-}$.

Supplementary Data S2. Gene ontology analysis of DEGs between WT NF54 and *Pfsrpk1*$^{-}$.

Supplementary Data S3. Summary transcripts where splicing has changed in *Pfsrpk1*$^{-}$parasites compared to parental parasites WT NF54.

Supplementary Data S4. Gene ontology enrichment analysis of DEGs with altered splicing.

Supplementary Figures S1-S4.

Supplementary Table 1. Oligonucleotides used in the study.

Supplementary Figures and Figure legends:

Figure S1.

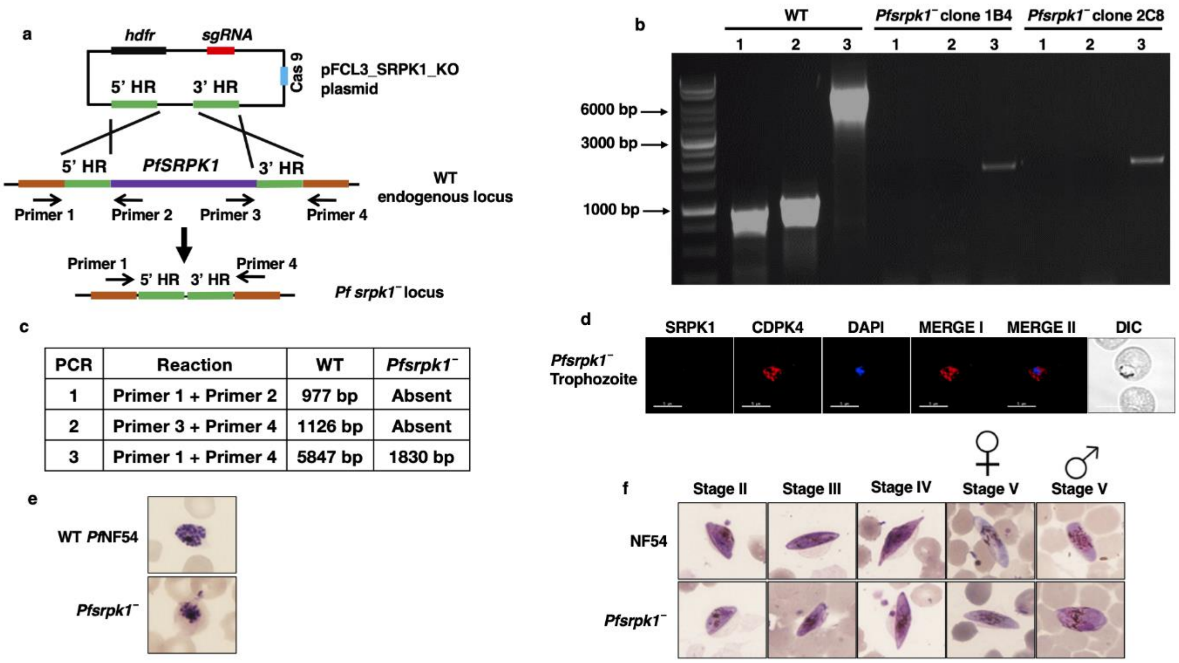

Supplementary Figure S1. Disruption of the *PfSRPK1* locus via CRISPR/Cas9. (**A**) The schematic shows the strategy for disrupting the *PfSRPK1* locus. pFC_SRPK1_KO plasmids has homology regions from 5’ (5’HR) and 3’ (3’HR) of *PfSRPK1* locus, single guide RNA seq (sgRNA) and human dihydrofolate reductase (hDHFR) locus and Cas9 cloned. (**B**) Confirmation of *PfSRPK1* deletion by diagnostic PCR. The oligonucleotides were designed from outside 5’HR and 3’HR and *PfSRPK1* locus and positions are

indicated by arrows in (**A**). (**C**) The expected sizes of amplicons for different set of PCRs performed in (**B**) are indicated. (**D**) IFAs were performed on *Pfsrpk1*$^{-}$ asexual stages (trophozoite) using thin smears using anti-*Pf*SRPK1 antisera (in green) in combination with anti-*Pf*CDPK4 (in red). *Pf*SRPK1 staining was negative for *Pfsrpk1*$^{-}$. (**E**) Light microscopy of Giemsa-stained thin smears for the development of WT *Pf*NF54 and *Pfsrpk1*$^{-}$ schizont stages showing daughter merozoites. 1,000×magnifcation. Representative Giemsa-stained images of WT *Pf*NF54 and *Pfsrpk1*$^{-}$ schizonts which were used for quantitative assessment of daughter merozoite numbers (**in Fig. 2B**) are shown. (**F**) WT *Pf*NF54 and *Pfsrpk1*$^{-}$ parasites were tested for their potential to form gametocytes. Light microscopy of Giemsa-stained thin smears showing development of WT *Pf*NF54 and *Pfsrpk1*$^{-}$ gametocytes and the four (II-V) distinct morphological stages. 1,000×magnifcation. Symbols for male and female gametocytes are shown on top of stage V gametocytes. Representative Giemsa-stained images of WT *Pf*NF54 and *Pfsrpk1*$^{-}$ gametocytes including stage V gametocytes which were used for quantitative assessment (**in Fig. 2D**) are shown.

Figure S2

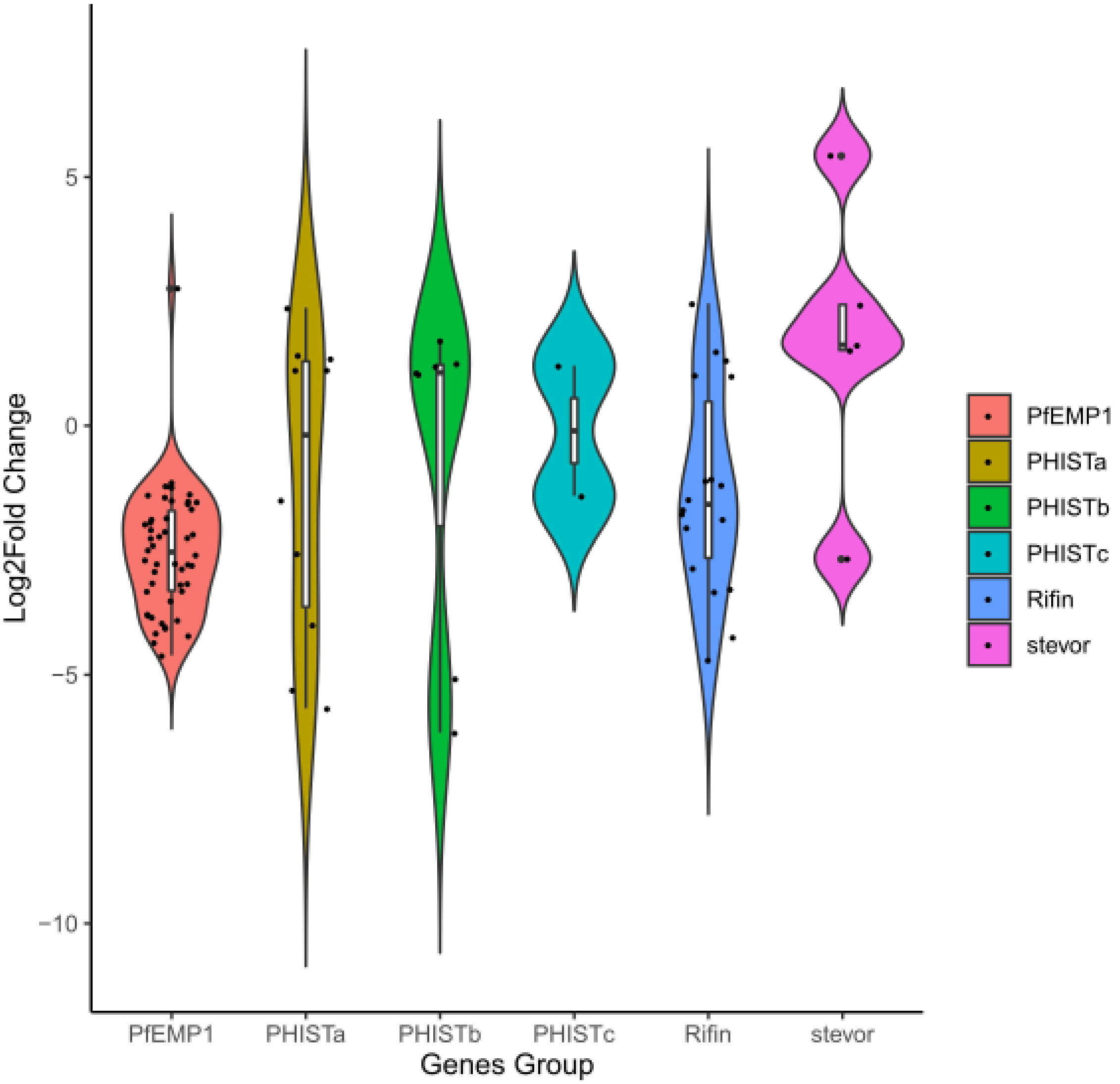

Supplementary Figure S2. Disruption of *PfSRPK1* results in altered transcription of genes encoded by multi-gene families associated with heterochromatin. Violin plot showing heterochromatin associated gene families with significant dysregulation in *Pfsrpk1*$^{-}$. Geometric mean fold-changes and p-values (two-sided, one sample t-test) are indicated.

Figure S3

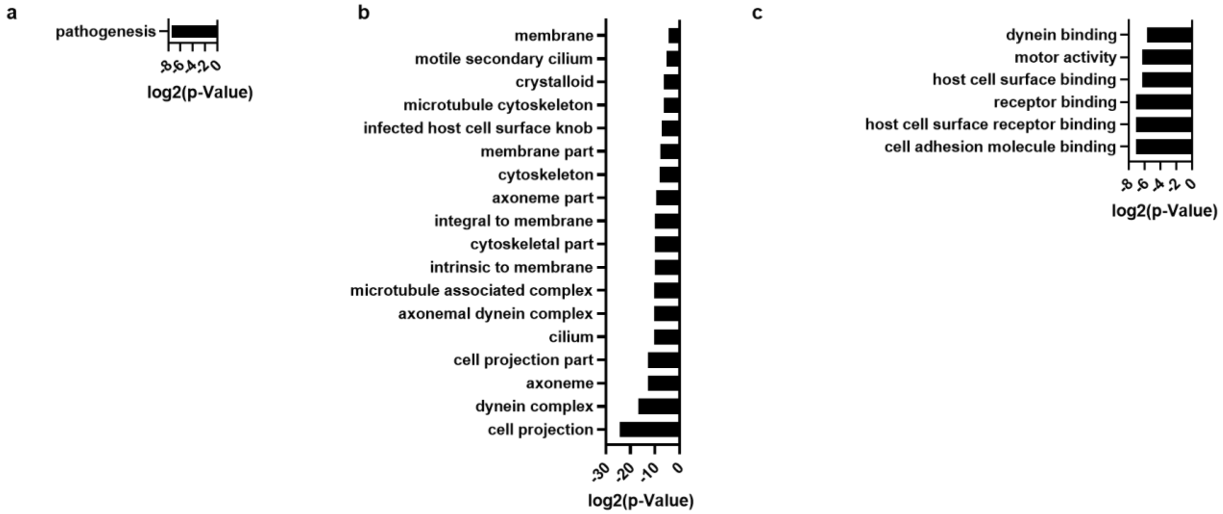

Supplementary Figure S3. Disruption of *PfSRPK1* results in dysregulation of splicing. (**A**) Biological process, (**B**) Cellular component, and (**C**) Molecular function gene ontology terms of the transcripts with altered splicing patterns are provided that highlight key biological processes that are impacted by *Pf*SRPK1 deletion. Log2(p values) are indicated on x-axis for all the categories.

Figure S4.

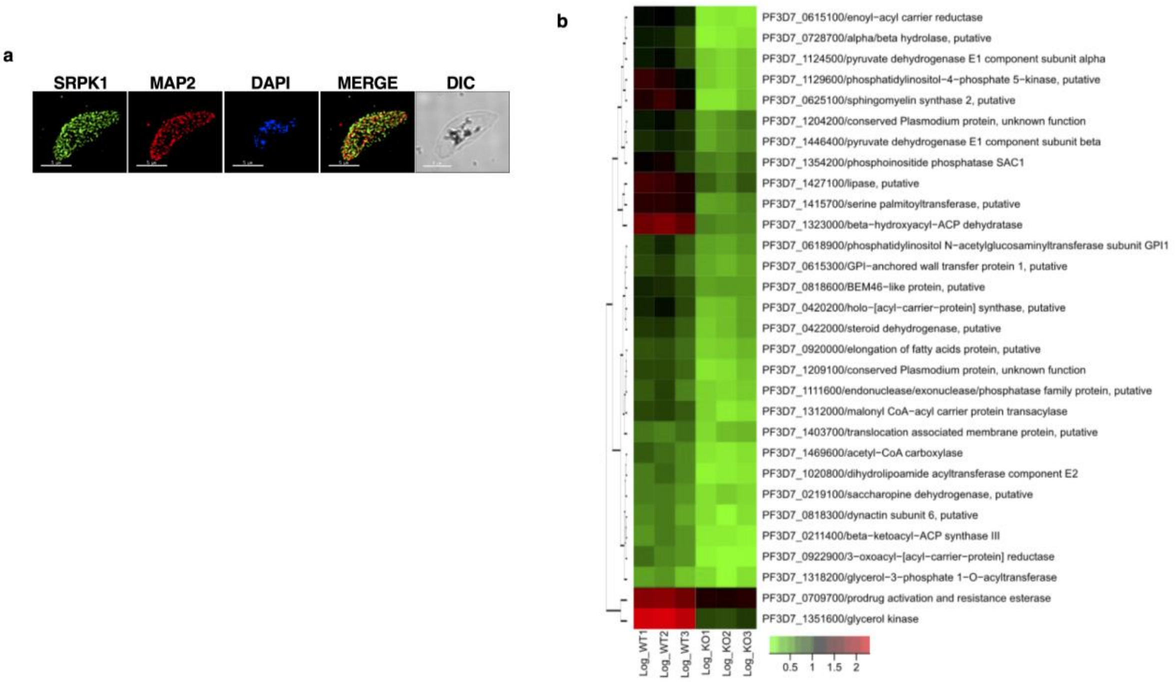

Supplementary Figure S4. Expression and localization of *Pf*SRPK1 and *Pf*MAP2 and altered transcription of genes encoding fatty acid metabolism in *Pfsrpk1*$^{-}$. (**A**) IFAs were performed on WT *Pf*NF54 stage V gametocytes using thin smears using anti-*Pf*SRPK1 antisera (in green) in combination with anti-*Pf*MAP2 (in red) to show coinciding expression. (**B**) Heatmaps showing DEGs encoding fatty acid metabolism enzymes in *Pfsrpk1*$^{-}$ gametocytes. Scale bar indicates Log2 fold change of TPM values of the samples in expression. Scale bar indicates Log2 fold change of TPM values of the samples in expression.

Supplementary Table 1. Oligonucleotides used in the study.

**Oligonucleotides used for generation of *Pfsrpk1*$^{-}$ parasites**	
**Oligo**	**Forward (5’-3’)**
PfSRPK1 5’Homo For	T**GCGGCCGC**GTAAAACTAGCGAAAAGAAAAAATGAGAAATAG
PfSRPK1 5’Homo Rev	CCAACCCGGGTATAGGCGCGCCTTATTAATATTTAATATAAAATTTGAGATTATATCCTG
PfSRPK1 3’Homo For	AGGCGCGCCTATACCCGGGTTGGTAGAATATATTTATTTATAGTCAAAATTTGCAAAAAA
PfSRPK1 3’Homo Rev	TAA**GTCGAC**GAACGTTGATTATAAAAAAGTATATATCGTTTCGTTTC
PfSRPK1 Guide 1 For	**TATT**AGTAGTGAAGATGCTACTTC
PfSRPK1 Guide 1 Rev	**AAAC**GAAGTAGCATCTTCACTACT
PfSRPK1 Guide 2 For	**TATT**GCGTCATTTAATGGTTTGTCT
PfSRPK1 Guide 2 Rev	**AAAC**AGACAAACCATTAAATGACGC
PfSRPK1 Geno5 For	TGAAGACAAACAATTTGTTTTATTATATACAGTTG
PfSRPK1 Geno5 Rev	TCCTTCGCAGTATTCATCACTTCCTTC
PfSRPK1 Geno3 For	CAAAAAATGCATATGAACGAAAAAACAAAC
PfSRPK1 Geno3 Rev	CCATTTGTTTAGTCTTTATTTTCAGTATTATGC
